# Internet Use Among Older Adults: Association With Health Needs, Psychological Capital, and Social Capital

**DOI:** 10.2196/jmir.2333

**Published:** 2013-05-16

**Authors:** Namkee G Choi, Diana M DiNitto

**Affiliations:** ^1^The University of Texas at AustinSchool of Social WorkThe University of Texas at AustinAustin, TXUnited States

**Keywords:** older adults, Internet use, health needs, psychological capital, social capital

## Abstract

**Background:**

Previous studies have identified socioeconomic status and health status as predictors of older adults’ computer and Internet use, but researchers have not examined the relationships between older adults’ health needs and psychological capital (emotional well-being and self-efficacy) and social capital (social integration/ties and support networks) to different types of Internet use.

**Objective:**

This study examined (1) whether older adults’ health conditions and psychological and social capital differentiate Internet users from nonusers, and (2) whether the Internet users differed in their types of Internet use on the basis of their health conditions and psychological and social capital.

**Methods:**

Data for this study came from the National Health and Aging Trends Study, which is based on a nationally representative sample of US Medicare beneficiaries aged 65 years and older. The sample for this study were those who resided in the community in their own or others’ homes (N=6680). Binary logistic regression analysis was used to compare health needs, psychological capital, and social capital among (1) any type of Internet users and nonusers, (2) Internet users who engaged in health-related tasks and Internet users who did not, (3) Internet users who engaged in shopping/banking tasks and Internet users who did not, and (4) Internet users only used the Internet for email/texting and all other Internet users.

**Results:**

Depressive and anxiety symptoms, measures of psychological capital, were negatively associated with Internet use among older adults (odds ratio [OR] 0.83, 95% CI 0.70-0.98, *P*=.03 and OR 0.79, 95% CI 0.65-0.97, *P*=.03, respectively), whereas most measures of social capital were positively associated with Internet use. Having more chronic medical conditions and engaging in formal volunteering increased the odds of Internet use for health-related tasks by 1.15 (95% CI 1.08-1.23, *P*<.001) and 1.28 (95% CI 1.05-1.57, *P*=.02), respectively, but anxiety symptoms decreased the odds (OR 0.74, 95% CI 0.55-0.99, *P*=.05). Religious service attendance was negatively associated with Internet use for shopping/banking activities (OR 0.75, 95% CI 0.62-0.91, *P*=.01). Anxiety symptoms increased the odds of using the Internet only for emails/texting (OR 1.75, 95% CI 1.12-2.75, *P*=.02), but formal volunteering decreased the odds (OR 0.63, 95% CI 0.43-0.92, *P*=.02). Other correlates of Internet use solely for emails/texting were older age (80-84 years and ≥85 years), a black or “other” racial/ethnic background, a high school education or less than high school, and lower income.

**Conclusions:**

The findings point to the importance of social capital in facilitating older adults’ learning and adoption of Internet technology. Older adults who used the Internet for email/texting purposes only were the most socially and economically disadvantaged group of Internet users. Computer/Internet training for older adults and computer/Internet use for various purposes need to consider the significant role their social capital can play.

## Introduction

Over the past 2 decades, Internet technology has increased access to health-related and non-health-related information and facilitated communication and social connections transcending geographic distance at relatively low cost. Almost 3 out of 4 people aged 65 years and older, compared to fewer than 1 out of 5 people aged between 20 and 44 years, have multiple (2 or more) chronic conditions [1]. Older adults are also more likely to feel socially isolated than younger adults, and social disconnectedness and perceived isolation are independently associated with lower levels of self-rated physical health and higher odds of having a mental health problem [2-4]. Older adults who have health problems and feel socially isolated are especially likely to benefit from using Internet technology because it allows them to carry out an increasingly diverse array of tasks, especially when they lack family, friends, and health and social service providers who can help with these tasks. Previous studies have documented the multiple benefits of computer and Internet use training for older adults [5,6]. Older adult Internet users note increased communication with those in their social networks; maintenance of geographically dispersed connections; convenience and benefits of searching for and increased learning from health-related information; increased ability to research non-health-related information, read news/magazines/books, and engage in continuing education activities; increased awareness of and connection to interest/support/hobby groups, events, and resources in their immediate and global communities; convenience of online shopping, banking, travel arrangements, and related information; and use of computer- and Internet-based entertainment [7-13].

Despite their rapidly increasing rate of Internet use, older adults still lag behind younger adults in Internet use in their everyday life [14,15]. An August 2012 Pew Internet Research Center survey found that only 58% of older adults (aged ≥65 years) in the United States are connected to and use the Internet, compared with 85% of those in the 50 to 64 age group and over 90% of those in the 18 to 49 age group [15]. Studies that examined determinants of Internet use found that older adults who were younger, non-Hispanic white, and of higher socioeconomic status (based on education, health literacy, and income) were more likely to use the Internet [14,16-18]. The significance of socioeconomic status as a correlate of Internet use is not surprising, because those of higher socioeconomic status have both the human and financial capital needed for adoption of rapidly changing technology. The cost of computer equipment and Internet connectivity poses a barrier to Internet use among low-income older adults [19].

Previous studies have also found that better overall health is positively associated with older adults’ computer and Internet use [14,18,20]. Some physical and functional health problems can pose barriers to computer use, such as difficulty performing activities of daily living (ADL) and instrumental activities of daily living (IADL), motor skills deficit due to arthritis and Parkinson’s disease, and vision impairment [21,22]. However, health problems/needs (eg, number of diagnosed chronic medical conditions, diagnosis of dementia/Alzheimer’s disease, and the number of ADL/IADL impairments) may also motivate older adults to take advantage of health information technology because some sicker individuals (eg, those with cancer) are more likely than their healthier peers to seek health information online in conjunction with a doctor visit [23]. In addition, older adults with health problems may use the Internet to manage various aspects of health care and address their daily health and disability care needs (eg, ordering/refilling prescriptions and contacting medical providers).

In addition to socioeconomic status and health care needs, psychological capital (eg, depressive symptoms, general anxiety symptoms, and general self-efficacy) and social capital (eg, indicators of social integration/ties and social support) are likely to influence older adults’ Internet use. With respect to psychological capital, previous research suggests that older adults are not afraid or unwilling to use technology and can acquire necessary skills; however, many older adults reported usability problems (eg, small fonts, difficulty of navigation) and associated frustration with the systems due in part to the cognitive, perceptual, and motor skill demands they experienced [9,10,24,25]. Older adults often express more anxiety about their ability to use these systems and less confidence in their ability to use them successfully than younger ones [9,26,27]. Older adults who positively perceived the Internet’s usefulness, ease of use, and efficacy and were more open to experiencing the Internet were more likely to be Internet users [23,28]. Werner et al [18] also found that older adults with an active coping style or a dispositional proactive approach to challenges (including learning to use technology in later life) were more likely to use a computer. However, they did not find depressive symptoms to be associated with older adults’ computer use, although another study found that older caregivers’ sense of social isolation and depressive symptoms abated when they participated in an online social interaction intervention [29].

On a macro level, social capital refers to the larger political and societal structures that promote a general sense of social cohesion, embeddedness, and trust, whereas microlevel social capital refers to individual resources that emerge from one’s social networks: social integration/ties and social support [30]. An individual’s social networks are the conceptual and structural core of his or her social capital. Those with strong and dense social networks have an easier and safer time accessing information because network members provide bridging and bonding support and contribute to boosting confidence and trust. When applied to older adults’ Internet use, those with a larger social network (eg, children, friends, volunteering buddies) are more likely to receive encouragement to learn to use the Internet and emotional and instrumental assistance in doing so by their social network members. Participation in activities with family, friends, and other network members is also likely to increase the need for and perceived usefulness of Internet connectivity as a means to maintain social integration and ties. One exploratory qualitative study of social capital and Internet use among older Australians (N=30) found that the most frequent pathways to use were having observed and talked with children and grandchildren about their computer/Internet use and having informal help from family and friends on how to use a computer and the Internet [11]. Study participants also mentioned that the need to use email to communicate with friends and relatives was a major reason for their Internet use, although they noted that online communication did not replace face-to-face contact. Moreover, the participants stated that online communication was responsible for expanding their network of close relationships and often led to face-to-face interactions that would not otherwise have occurred [11]. One study of US older adults in the Detroit area found that older adult computer users, as compared to nonusers, were more likely to be employed, have memberships in community organizations, and do volunteer work [31]. Another study also found that older adults who tended to be actively involved in the community, rather than withdrawn or behaviorally disengaged, were more likely to use computers [18].

A literature review on the determinants of older adults’ Internet use reveals gaps in 2 areas. First, although the Internet is used for an increasing variety of tasks including personal communication, commerce, information seeking, social networking, job searches, and entertainment, no studies have examined the factors associated with different types of Internet use. Previous studies did find that Internet use differences are affected by the level of Internet operational and other digital skills (eg, operating an Internet browser; using Internet-based search engines; completing forms on the Internet; navigating various types of websites; locating specific, detailed, or customized information; and evaluating the source and the quality of information), which was positively associated with education, but negatively associated with age [32-34]. A survey of Internet use showed that email and information searches tend to be the most common uses among both older and younger adults [35]. However, because older adults use the Internet for a diverse array of activities, Internet use differences among older adults are likely to be influenced by different characteristics. Second, although some previous studies found that psychological and social resources significantly influenced Internet use, the samples studied tended to be small. The relationships among Internet use and psychological and social capital have not yet been tested with a large, nationally representative sample of older adults. The examination of psychological and social capital on different types of Internet use is especially important given that older adults may require these resources to use the Internet for activities that require advanced skills. Older adults who are disadvantaged in terms of psychological and social capital may be more likely to use the Internet for limited purposes only.

Using a nationally representative sample of older adults aged 65 years and older in the United States, the present study had 2 purposes. The first was to examine whether health conditions and psychological and social capital resources differentiate Internet users from nonusers. The second was to determine whether the subgroup of Internet users differed in their types of Internet use based on their health conditions and psychological and social capital. Controlling for demographic and socioeconomic factors, the study hypotheses were (H1) older adults with symptoms of depression and/or anxiety will be less likely to use the Internet, (H2) older adults with higher levels of general self-efficacy will be more likely to use the Internet, (H3) older adults with at least 1 living child and/or sibling will be more likely to use the Internet, (H4) older adults with a higher level of social integration/ties from engagement in paid work activities, formal volunteer work, informal caregiving, and an active social life will be more likely to use the Internet, (H5) older adult Internet users with a higher levels of physical and functional health needs will be more likely to use the Internet for health-related tasks, (H6) older adult Internet users with higher levels of psychological and social capital will be more likely to use the Internet for health-related tasks and shopping/banking activities, and (H7) older adult Internet users with lower levels of psychological and social capital will be more likely to use the Internet for email/texting only. This is the first study to try to identify characteristics associated with different Internet activities using a nationally representative sample of older adults in the United States. The findings contribute to understanding the role of older adults’ psychological and social capital on their Internet use and shed further light on significant disparities in Internet access and use among older adults.

## Methods

### Data Source and Sample

This study analyzed secondary data drawn from the first interview wave of the National Health and Aging Trends Study (NHATS). The National Institute on Aging supports NHATS under a cooperative agreement with the Johns Hopkins University Bloomberg School of Public Health. Westat, a statistical survey organization headquartered in Rockville, Maryland, collected the data. The NHATS is intended to be a new resource for the scientific study of physical, psychological, and social functioning in later life and is based on a nationally representative sample of US Medicare beneficiaries aged 65 years and older (N=8077) who resided in the community in their own or another’s home or in residential care settings, including nursing homes and other facilities [36]. Face-to-face individual interviews, lasting approximately 2 hours, were administered in 2011 by Westat’s professionally trained interviewers with sample persons in all settings (except nursing homes) to collect detailed information on activities of daily life, living arrangements, general and technological environment of the home, health conditions, work status and participation in valued activities, mobility and use of assistive devices, cognitive functioning, and help provided with daily activities (self-care, household, and medical), economic status, and well-being. The NHATS sample design was age-stratified so that persons were selected from 5-year age groups between the ages of 65 and 90, and from persons age 90 and older. Persons in older age groups and persons whose race was listed as black on the Center for Medicare and Medicaid Services enrollment file were oversampled [37]. Detailed data collection procedures and variable definitions are described in the NHATS User Guide [38]. The analyses in this study included only those sample persons (N=6680) who resided in their own or another’s home, and excluded those in residential care settings, such as nursing homes (n=468) or other such settings (n=412), and those represented by proxy respondents, such as their spouse or child (n=517) due to dementia, illness, hearing impairment, and/or speech impairment. These exclusions were based on both systematic and respondent-level missing data on many variables (eg, psychological and social capital resources) included in this study.

### Measures

#### Internet Use

Each NHATS sample participant was asked if he or she had a working cell phone and a working desktop or laptop computer at home (response categories: yes, no, yes but doesn’t know how to use a computer, refused, and don’t know). Those who did not have a computer at home were asked if they used a computer anywhere else (eg, in the building where they lived, at a library, and/or at a friend’s or family member’s home) in the past month. Those with a cell phone and/or who used a computer were asked if they ever (1) sent messages by email or via texting (described as “texting is like email but usually done on a phone”); (2) went on the Internet or online for any other reason than email or texting (“In the last month, besides email or texting, have you ever gone on the Internet or online for any other reason?”); (3) went on the Internet or online to contact a medical provider (to make or change medical appointments, get test results, request referrals or prescriptions, or get advice), handle Medicare or other health insurance matters (going to Medicare’s website or another insurer’s website to find out what is covered, compare plans or providers, find out about bills, or file a claim), and get information about their health conditions; and (4) went on the Internet or online to shop for groceries or personal items, pay bills or do banking, and order or refill prescriptions. The response categories were yes, no, refused, and don’t know. The time frame for the Internet/online use for all health-related tasks, except ordering or refilling prescriptions, was within the past year, and for all other activities was within the past month. Note that NHATS used the term “Internet or online” interchangeably in all questions, without distinguishing between them.

In the present study, Internet use was grouped into 4 types: (1) any health-related tasks (ordering or refilling prescriptions, contacting medical providers, handling Medicare or other health insurance matters, and/or get information on health conditions) with or without other types of Internet use; (2) shopping for groceries or personal items, paying bills, and/or banking (shopping/banking hereafter) with or without other types of Internet use; (3) email/texting with or without other types of Internet use (with the focus of multivariate analysis on email/texting only without any other type of Internet use); and (4) any other tasks that do not fall under any of the other 3 categories of tasks. This fourth category was derived from comparing those who reported Internet use but did not report any email/texting, health-related tasks, or shopping/banking.

#### Demographic and Socioeconomic Factors

Demographic and socioeconomic variables included age group (65-69 years [reference group], 70-74 years, 75-79 years, 80-84 years, and ≥85 years); gender (female or male); race/ethnicity (non-Hispanic white [reference group], non-Hispanic black, Hispanic, and all others); living arrangement (living with spouse vs all others); level of education (less than high school/don’t know/refused, high school diploma or GED, some college or an associate’s degree, and bachelor’s degree or higher [reference group]); and total income (in units of $10,000 for multivariate analysis). Missing values in education level due to respondents’ uncertainty (don’t know) or refusal to answer were grouped with the “less than high school” category based on multiple bivariate analyses of other sample characteristics (eg, Internet use, sociodemographics, health conditions, and psychological/social capital) that showed similarities between the don’t know or refused to answer group (unweighted n=67) and the less than high school group.

#### Health Needs

Health needs included the number of chronic medical conditions diagnosed by a doctor (including high blood pressure, heart attack/heart disease, arthritis, osteoporosis, diabetes, lung disease, stroke, and cancer), diagnosis of dementia or Alzheimer’s disease (yes vs no), and the number of ADL/IADL impairments. ADLs included eating, bathing, toileting, dressing, getting in and out of bed, getting in and out of a chair, and walking inside. IADLs included preparing meals, doing laundry, doing light housework, shopping for groceries, managing money, taking medication, and making telephone calls. Separate questions were used to collect data on the diagnosis of each medical condition and each ADL/IADL impairment, and the numbers of diagnoses and the functional impairments were added up, respectively. The small number of missing values (don’t know or refused to answer) for some of the medical conditions and ADL/IADL variables were treated as an absence of a diagnosis or impairment to arrive at conservative estimates.

#### Psychological Capital

Psychological capital included symptoms of depression and anxiety and general self-efficacy. For depression, each sample person was asked how often in the past month he or she (1) had little interest or pleasure in doing things, and (2) felt down, depressed, or hopeless. For anxiety, participants were asked how often in the past month he or she (1) felt nervous, anxious, or on edge, and (2) had been unable to stop or control worrying. The response categories were 1=not at all, 2=several days, 3=more than half the days, and 4=nearly every day. In the present study, those who responded with more than half the days to either item for depression or anxiety were categorized as depressed or anxious, respectively. To create a general efficacy score, the responses to the following 4 questions were summed: (1) I feel confident and good about myself, (2) I gave up trying to improve my life a long time ago (reverse-coded), (3) When I really want to do something, I usually find a way to do it, and (4) I have an easy time adjusting to change. The response categories were 1=agree not at all, 2=agree a little, and 3=agree a lot. The Cronbach alpha (internal consistency reliability of the 4 items measuring general efficacy) was low at .57, most likely because of the small number of items in the scale. According to the NHATS User Guide [33], these psychological capital variables draw on items similar to those used in Midlife in the United States: A Study of National Health and Well-being (MIDUS), with changes in the reference period as “last month” and fewer response categories.

#### Social Capital

Social capital was measured by whether or not the sample person reported (1) having at least 1 living child/stepchild; (2) having at least 1 living sibling; and whether in the past month he or she ever (3) worked for pay or owned a business; (4) did any volunteer work; (5) cared for or looked after an adult or child who could not care for themselves; (6) visited in person with friends or family not living with them either at your home or theirs; (7) attended religious services; (8) participated in clubs, classes, or other organized activities; and (9) went out for enjoyment, including going to dinner, to a movie, to gamble, or to hear music or see a play. Response categories for all these variables were yes, no, refused, and don’t know. The small number of missing values (don’t know and refused) in any social capital variable were treated as absence of the condition or activity to arrive at conservative estimates.

### Analysis Strategy

Univariate frequency analysis was used to examine demographic and socioeconomic characteristics, health needs, psychological capital, social capital, cell phone and computer ownership, and any Internet use among all sample persons, and the type(s) of Internet use among Internet users. Bivariate analyses, using chi-square and *t* tests, were used to compare Internet users and nonusers on relevant variables. Hypothesis testing was conducted by using binary logistic regression analysis to compare (1) any type of Internet users with nonusers, (2) Internet users who engaged in health-related tasks with Internet users who did not, (3) Internet users who engaged in shopping/banking tasks with Internet users who did not, and (4) Internet users whose sole use was for email/texting with all other Internet users. Although users that did not engage in Internet use for health-related tasks, shopping/banking, and email/texting were identified, this group was excluded from the multivariate analysis because of the unspecified and possibly widely varying nature of their Internet activities. Because of the cross-sectional nature of the data, the relationships examined are correlational, not causal. Analyses were conducted with svy commands in Stata 12 (StataCorp LP, College Station, Texas, USA) to account for the NHATS’ complex multistage, stratified sampling design [37].

## Results

### Computer Ownership and Internet Use

As seen in Table 1, 80.19% of the study sample had a working cell phone, 64.4% had a computer at home and knew how to use it, 4.11% had a computer at home but did not know how to use it, and 2.20% did not have a computer at home but used one outside their home. Of the study sample, 50.60% reported that they went on the Internet or online for at least 1 purpose in the past month, whereas 49.40% did not report any Internet/online use, such as email/texting or carrying out other activities. Of the computer users, 25.42% did not go online for email/texting or any other type of Internet use in the past month. Of those who went on the Internet/online for email/texting or any other tasks, only 1.80% reported that they did not use a computer, implying that these people may have relied exclusively on cell phones and/or other devices.

The results also show the prevalence of different kinds of tasks that these community-dwelling older adults conducted on the Internet/online: 43.35% sent emails or text messages, 20.74% paid bills and did banking, 16.83% searched information on health conditions for self or others, 14.9% shopped for groceries or personal items, 8.41% ordered or refilled prescriptions, 7.45% contacted medical providers, and 5.64% handled Medicare or other health insurance matters. Among the Internet/online users, 85.68% sent emails or text messages, 40.99% paid bills and did banking, 33.26% searched information on health conditions for self or others, 29.40% shopped for groceries or personal items, 16.62% ordered or refilled prescriptions, 14.72% contacted medical providers, and 11.15% handled Medicare or other health insurance matters. In summary, 45.15% of the Internet/online users conducted health-related tasks, 51.23% paid bills, did banking, and/or shopped for groceries or personal items—common tasks related to everyday life—and 30.90% conducted activities pertaining to all these categories of Internet use. The findings also show that 8.94% used the Internet/went online solely for sending emails or text messages, and 8.76% used it for purposes/tasks other than emailing/texting, health-related tasks, and the tasks related to everyday life specified previously. The NHATS did not ask about these other Internet/online activities. However, based on a recent Pew survey of the Internet activities in which US adults engage [34], these other activities may include searching for information on a hobby or interest or a map or driving directions, checking weather, making travel reservations, getting news or information about sports, participating in social networking/dating programs, and so forth.

### Comparison Between Internet Users and Nonusers

Table 2 shows that the younger age groups (65-69 years and 70-74 years) were disproportionately (more highly) represented among the Internet users, whereas the older age groups (75-79 years, 80-84 years, and ≥85 years) were disproportionately (more highly) represented among the nonusers. Men, non-Hispanic whites, those married and living with their spouse, and those with at least some college education were also overrepresented among the Internet users. The median income of the Internet users was more than twice that of the nonusers. The Internet users were in better health than the nonusers. With respect to psychological capital, the rates of depression and anxiety symptoms among the Internet users were half of those among the nonusers. The Internet users also had higher self-efficacy scores. With respect to social capital, a higher proportion of the Internet users than nonusers had at least 1 child, but the 2 groups were equally likely to have at least 1 sibling. The Internet users were significantly more advantaged than nonusers in all other indicators of social integration/ties and social support.

**Table 1:** Cell phone and computer ownership and Internet/online use of older adults in the United States (N=6680).

Internet use patterns		%^a^
**Technology equipment among all sample persons**		
	A working cell phone	80.19
	A working computer at home and knew how to use	64.39
	A computer at home but did not know how to use	4.11
	No computer at home but used it elsewhere last month	2.20
**Type of Internet use among all sample persons**		
	For any purpose (listed below and other tasks)	50.60
	To send emails or text messages	43.35
	To order or refill prescriptions	8.41
	To contact any medical provider	7.45
	To handle Medicare or other health insurance matters	5.64
	To obtain information about health conditions for self or others	16.83
	To pay bills or do banking	20.74
	To shop for groceries or personal items	14.88
**Type of Internet use among any Internet users (n=3380** ^a^ **)**		
	To send emails or text messages	85.68
	To order or refill prescriptions	16.62
	To contact any medical provider	14.72
	To handle Medicare or other health insurance matters	11.15
	To obtain information about health conditions for self or others	33.26
	To pay bills or do banking	40.99
	To shop for groceries or personal items	29.40
**Summary of different types of Internet use among any Internet users (n=3380** ^a^ **)**		
	To send emails or text messages	85.68
	For emails or texting purposes only (not any other use)	8.94
	To conduct health-related tasks (order/refill prescriptions, contact medical provider, handle insurance matters, and/or obtain health information)	45.15
	To pay bills, do banking, and/or shop for groceries or personal items	51.23
	To conduct all of the above activities (emails/text messages, health-related tasks, and banking/shopping)	30.90
	For other purposes than any of the above	8.76

^a^Weighted.

**Table 2:** Sample characteristics: weighted statistics.

Demographics/socioeconomic status		All (N=6680, 100%)	No Internet use (n=3300, 49.40%)	Any Internet use (n=3380, 50.60%)	*P* value^a^
**Age group (years)**					<.001
	65-69	30.13	18.63	41.36	
	70-74	26.17	22.70	29.56	
	75-79	19.40	23.25	15.64	
	80-84	14.16	19.16	9.28	
	≥85	10.14	16.26	4.15	
**Gender**					<.001
	Male	44.20	41.14	47.19	
	Female	55.80	58.86	52.81	
**Race/ethnicity**					<.001
	Non-Hispanic white	80.85	73.09	88.42	
	Non-Hispanic black	7.97	11.51	4.53	
	Hispanic	6.73	10.19	3.35	
	All other/don’t know/refused	4.45	5.21	3.71	
**Marital status**					<.001
	Married/cohabiting	59.64	49.87	69.18	
	Widowed	24.86	12.50	12.11	
	Divorced/separated	12.30	33.46	16.47	
	Never married	3.19	4.18	2.24	
Live with spouse		58.69	48.61	68.52	<.001
**Education**					<.001
	Less than high school/don’t know/refused	21.43	36.50	6.71	
	High school diploma or GED	27.08	33.94	20.38	
	Some college or associate degree	26.61	19.93	33.13	
	Bachelor’s degree or higher	24.88	9.62	39.78	
Income ($), median		32,000	21,511	50,000	
**Health conditions/needs**					
	Chronic medical conditions, mean (SE)	2.27 (0.03)	2.44 (0.03)	2.11 (0.03)	<.001
	Dementia/Alzheimer’s disease, %	2.05	3.58	0.01	<.001
	ADL/IADL impairments, mean (SE)	1.03 (0.06)	1.44 (0.06)	0.64 (0.07)	<.001
**Psychological capital**					
	Depressive symptoms last month	16.28	21.89	10.80	<.001
	Anxiety symptoms last month	13.19	18.30	8.19	<.001
	Efficacy score, mean (SE)	10.71 (0.03)	10.44 (0.04)	10.97 (0.03)	<.001
**Social capital**					
	Have at least 1 living child	88.21	86.06	90.31	<.001
	Have at least 1 sibling	80.00	79.47	80.51	.30
	Worked for pay last month	19.63	10.77	28.28	<.001
	Did formal volunteering last month	26.33	17.37	35.07	<.001
	Provided informal caregiving last month	20.00	16.78	23.14	<.001
	Visited family/friend last month	88.24	83.44	92.92	<.001
	Attended religious service last month	57.69	56.30	59.04	.02
	Participated in clubs/classes/other organized activities last month	38.12	25.51	50.43	<.001
	Went out for enjoyment last month	80.48	70.28	90.44	<.001

^a^ *P* values denote difference between nonusers and users based on chi-square tests or independent samples *t* tests.

### Association Between Internet Use and Health Needs, Psychological Capital, and Social Capital

As seen in Table 3, controlling for demographic and socioeconomic variables, a diagnosis of dementia or Alzheimer’s disease was the only health status variable significantly associated with Internet use. Those individuals with these diagnoses were significantly less likely to use the Internet (odds ratio [OR] 0.37, 95% CI 0.20-0.65, *P*<.001). Depressive and anxiety symptoms were also negatively associated with the Internet use (depression: OR 0.83, 95% CI 0.70-0.98, *P*=.03; anxiety: OR 0.79, 95% CI 0.65-0.97, *P*=.03), but self-efficacy score was not. In terms of social capital resources, having a child (OR 1.47, 95% CI 1.18-1.84, *P*<.001), engagement in paid work (OR 1.65, 95% CI 1.34-2.03, *P*<.001) and formal volunteering (OR 1.33, 95% CI 1.09-1.62, *P*=.01), friend/family visits (OR 1.25, 95% CI 1.03-1.52, *P*=.02), attending organized activities (OR 1.73, 95% CI 1.46-2.05, *P*<.001), and participating in entertainment activities (OR 1.69, 95% CI 1.36-2.08, *P*<.001) were all positively associated with Internet use, whereas having a sibling (OR 0.81, 95% CI 0.68-0.97, *P*=.02) and attending religious services (OR 0.82, 95% CI 0.73-0.94, *P*=.01) were significantly negatively associated with use. Informal caregiving was not a significant correlate.

Age group, race/ethnicity, level of education, living arrangement, and total income were significantly associated with Internet use versus nonuse. Compared with the 65 to 69 age group, older age groups had significantly lower odds of Internet use (80-84 years: OR 0.21, 95% CI 0.18-0.26, *P*<.001; ≥85 years: OR 0.12, 95% CI 0.09-0.16, *P*<.001). Compared with non-Hispanic whites, non-Hispanic blacks and Hispanics had significantly lower odds of Internet use (non-Hispanic black: OR 0.41, 95% CI 0.33-0.51, *P*<.001; Hispanic: OR 0.55, 95% CI 0.39-0.78, *P*<.001). Compared to those with at least a bachelor’s degree, those with less than a high school education or who refused to reveal their level of education had Internet use odds of 0.08 (95% CI 0.06-0.11, *P*<.001), those with high school diploma or General Education Development (GED) had odds of 0.18 (95% CI 0.14-0.24, *P*<.001), and those with some college education had odds of 0.46 (95% CI 0.37-0.57, *P*<.001). Compared to not living with a spouse, those living with a spouse had higher odds of Internet use (OR 1.38, 95% CI 1.19-1.59, *P*<.001). *F*_28,29_ was 37.65 (*P*<.001).

### Association Between Different Types of Internet Use and Health Needs, Psychological Capital, and Social Capital

As Table 4 shows, Internet use for health-related tasks among older adults was significantly associated with the number of chronic medical conditions. Having a greater number of chronic medical conditions was associated with a higher likelihood (OR 1.15, 95% CI 1.08-1.23, *P*<.001) of using the Internet for health-related tasks as opposed to other tasks. However, a diagnosis of dementia/Alzheimer’s disease and the number of ADL/IADL impairments were not significant correlates. Anxiety symptoms was associated with a lower likelihood (OR 0.74, 95% CI 0.55-0.99, *P*=.05). Depressive symptoms and self-efficacy were not significant correlates. Among social capital variables, formal volunteering was significantly positively associated with Internet use for health-related tasks (OR 1.28, 95% CI 1.05-1.57, *P*=.02). Age group, race/ethnicity, level of education, living arrangement, but not total income, were significant correlates of Internet use for health-related tasks as opposed to other tasks. For example, non-Hispanic blacks, as opposed to non-Hispanic whites, had lower odds (0.70, 95% CI 0.53-0.92, *P*=.01) of Internet use for health-related tasks, whereas Hispanics and the “other” racial/ethnic group did not differ significantly from non-Hispanic whites. *F* tests for health-related tasks, shopping/banking, and email/texting only were *F*_28,29_=7.62 (*P*<.001), *F*_28,29_=6.60 (*P*<.001), *F*_28,29_=4.56 (*P*<.001), respectively.

**Table 3:** Correlates of any Internet/online use among all sample persons: logistic regression analysis results (N=6680).

Variable		Internet/online use vs no Internet use	
		OR (SE)	95% CI
**Age group (years)**			
	65-69	1.00	
	70-74	0.62 (0.07)^a^	0.49-0.78
	75-79	0.31 (0.03)^a^	0.25-0.38
	80-84	0.22 (0.02)^a^	0.18-0.26
	≥85	0.12 (0.02)^a^	0.09-0.16
**Gender**			
	Male	1.00	
	Female	0.97 (0.07)	0.83-1.12
**Race/ethnicity**			
	Non-Hispanic white	1.00	
	Non-Hispanic black	0.41 (0.04)^a^	0.33-0.51
	Hispanic	0.55 (0.09)^a^	0.39-0.78
	All other/don’t know/refused	0.86 (0.18)	0.56-1.32
**Education**			
	BA/BS or higher	1.00	
	Less than high school/don’t know/refused	0.08 (0.01)^a^	0.06-0.10
	High school diploma or GED	0.18 (0.02)^a^	0.14-0.24
	Some college or associate degree	0.46 (0.05)^a^	0.37-0.57
**Living arrangement**			
	Not living with a spouse	1.00	
	Living with a spouse	1.38 (0.10)^a^	1.19-1.59
Total income (in $10,000)		1.01 (0.01)	1.00-1.02
Number of chronic illnesses		1.01 (0.02)	0.97-1.05
**Dementia/Alzheimer’s disease**			
	No	1.00	
	Yes	0.37 (0.11)^a^	0.20-0.66
Number of ADL/IADL impairments		1.01 (0.02)	0.97-1.04
**Depression**			
	No	1.00	
	Yes	0.83 (0.07)^c^	0.70-0.99
**Anxiety**			
	No	1.00	
	Yes	0.79 (0.08)^c^	0.65-0.97
Self-efficacy		1.05 (0.03)	0.99-1.11
**Living child**			
	No	1.00	
	Yes	1.47 (0.16)^a^	1.18-1.84
**Living sibling**			
	No	1.00	
	Yes	0.81(0.07)^c^	0.68-0.99
**Worked for pay last month**			
	No	1.00	
	Yes	1.65 (0.17)^a^	1.34-2.03
**Did volunteering last month**			
	No	1.00	
	Yes	1.33 (0.13)^b^	1.09-1.62
**Informal caregiver last month**			
	No	1.00	
	Yes	1.00 (0.09)	0.83-1.19
**Visited family/friend last month**			
	No	1.00	
	Yes	1.25 (0.12)^c^	1.03-1.52
**Attended religious service last month**			
	No	1.00	
	Yes	0.82 (0.05)^c^	0.73-0.94
**Attended clubs/classes/organized activities last month**			
	No	1.00	
	Yes	1.73 (0.15)^a^	1.46-2.05
**Went out for enjoyment last month**			
	No	1.00	
	Yes	1.69 (0.18)^a^	1.36-2.08

^a^ *P*<.001

^b^ *P*<.01

^c^ *P*<.05

**Table 4:** Correlates of different types of Internet/online use among Internet/online users: logistic regression analysis results.

Variable		Health-related tasks^a^		Shopping/banking^b^		Email/texting only^c^	
		OR (SE)	95% CI	OR (SE)	95% CI	OR (SE)	95% CI
**Age group (years)**							
	65-69	1.00		1.00		1.00	
	70-74	0.76 (0.07)^e^	0.64-0.91	0.63 (0.07)^d^	0.50-0.79	1.25 (0.23)	0.87-1.82
	75-79	0.71 (0.07)^e^	0.58-0.88	0.64 (0.08)^e^	0.50-0.82	1.27 (0.22)	0.90-1.79
	80-84	0.63 (0.08)^d^	0.49-0.81	0.40 (0.06)^d^	0.30-0.55	2.60 (0.55)^d^	1.70-3.98
	≥85	0.66 (0.11)^f^	0.48-0.92	0.32 (0.05)^d^	0.24-0.43	3.17 (0.77)^d^	1.95-5.17
**Gender**							
	Male	1.00		1.00		1.00	
	Female	1.09 (0.10)	0.91-1.32	1.21 (0.12)	0.99-1.48	0.76 (0.12)	0.56-1.03
**Race/ethnicity**							
	Non-Hispanic white	1.00		1.00		1.00	
	Non-Hispanic black	0.70 (0.09)^f^	0.53-0.92	0.90 (0.13)	0.67-1.21	1.60 (0.35)^f^	1.03-2.49
	Hispanic	0.85 (0.20)	0.54-1.46	1.01 (0.25)	0.61-1.66	1.19 (0.45)	0.56-2.54
	All other/don’t know/refused	0.69 (0.16)	0.42-1.11	1.16 (0.23)	0.78-1.72	2.31 (0.63)^e^	1.33-3.99
**Education**							
	BA/BS or higher	1.00		1.00		1.00	
	Less than high school/don’t know/refused	0.39 (0.07)^d^	0.27-0.56	0.34 (0.06)^d^	0.24-0.50	2.20 (0.60)^e^	1.35-3.39
	High school diploma or GED	0.49 (0.06)^d^	0.38-0.63	0.45 (0.07)^d^	0.34-0.62	1.61 (0.31)^f^	1.09-2.38
	Some college or associate degree	0.68 (0.06)^d^	0.56-0.82	0.79 (0.08)^f^	0.64-0.98	1.02 (0.20)	0.69-1.51
**Living arrangement**							
	Not living with a spouse	1.00		1.00		1.00	
	Living with a spouse	1.23 (0.12)^f^	1.03-1.51	0.85 (0.08)	0.70-1.02	1.21 (0.21)	0.85-1.73
Total income (in $10,000)		1.00 (0.01)	0.99-1.00	1.00 (0.01)	0.99-1.00	0.93 (0.02)^e^	0.89-0.98
Number of chronic illnesses		1.15 (0.04)^d^	1.08-1.23	1.00 (0.03)	0.94-1.07	0.99 (0.05)	0.89-1.10
**Dementia/Alzheimer’s disease**							
	No	1.00		1.00		1.00	
	Yes	1.22 (0.71)	0.38-3.91	0.86 (0.50)	0.27-2.73	2.68 (1.98)	0.61-11.77
Number of ADL/IADL impairments		1.01 (0.02)	0.97-1.05	1.02 (0.02)	0.97-1.07	0.99 (0.03)	0.93-1.06
**Depression**							
	No	1.00		1.00		1.00	
	Yes	1.07 (0.20)	0.75-1.54	1.14 (0.17)	0.85-1.53	1.34 (0.33)	0.82-2.20
**Anxiety**							
	No	1.00		1.00		1.00	
	Yes	0.74 (0.11)^f^	0.55-0.99	0.78 (0.13)	0.56-1.09	1.75 (0.39)^f^	1.12-2.75
Self-efficacy		0.95 (0.04)	0.88-1.02	1.03 (0.04)	0.97-1.11	1.13 (0.07)	0.99-1.29
**Living child**							
	No	1.00		1.00		1.00	
	Yes	1.06 (0.17)	0.77-1.47	1.29 (0.20)	0.95-1.76	1.65 (0.42)	0.99-2.76
**Living sibling**							
	No	1.00		1.00		1.00	
	Yes	0.95(0.10)	0.77-1.18	0.97 (0.10)	0.79-1.20	0.97 (0.15)	0.72-1.32
**Worked for pay**							
	No	1.00		1.00		1.00	
	Yes	0.97 (0.09)	0.80-1.17	1.13 (0.10)	0.95-1.35	1.23 (0.24)	0.83-1.82
**Did volunteering**							
	No	1.00		1.00		1.00	
	Yes	1.28 (0.13)^f^	1.05-1.57	1.12 (0.12)	0.79-1.20	0.63 (0.12)^f^	0.43-0.92
**Informal caregiver**							
	No	1.00		1.00		1.00	
	Yes	1.07 (0.09)	0.91-1.27	1.10 (0.12)	0.89-1.35	0.74 (0.12)	0.53-1.02
**Visited family/friend**							
	No	1.00		1.00		1.00	
	Yes	1.26 (0.21)	0.91-1.74	1.10 (0.17)	0.80-1.51	1.04 (0.26)	0.63-1.72
**Attended religious service**							
	No	1.00		1.00		1.00	
	Yes	0.84 (0.07)	0.70-1.01	0.75 (0.7)^e^	0.62-0.91	1.35 (0.21)	0.99-1.84
**Attended clubs/classes/ organized activities**							
	No	1.00		1.00		1.00	
	Yes	1.15 (0.12)	0.93-1.42	1.08 (0.10)	0.89-1.31	0.75 (0.13)	0.53-1.06
**Went out for enjoyment**							
	No	1.00		1.00		1.00	
	Yes	1.10 (0.17)	0.81-1.50	1.10 (0.18)	0.79-1.53	0.71 (0.16)	0.45-1.10

^a^Coded as 1 for using Internet for any health-related task and 0 for not using the Internet for health-related task.

^b^Coded as 1 for using Internet for any shopping/banking task and 0 for not using the Internet for shopping/banking task.

^c^Coded as 1 for using Internet for email/texting purposes only and 0 for using the Internet for email/texting and other tasks (ie, health-related tasks and/or shopping/banking tasks).

^d^ *P*<.001.

^e^ *P*<.01.

^f^ *P*<.05.

Internet use for shopping/banking activities among older adults was significantly negatively associated with religious service attendance only (OR 0.75, 95% CI 0.62-0.91, *P*=.01). None of the health needs and psychological and other social capital variables was correlated with this type of Internet use. Of the demographic and socioeconomic status variables, age group and education level were significant correlates, whereas gender, race/ethnicity, and income were not significant correlates.

Internet use solely for sending emails or text messages was significantly positively associated with anxiety symptoms (OR 1.75, 95% CI 1.12-2.75, *P*=.02), but it was significantly negatively associated with engagement in formal volunteering (OR 0.63, 95% CI 0.43-0.92, *P*=.02). Compared to those in the 60 to 65 age group, those in the 80 to 84 year and 85 year and older age groups had significantly higher odds (80-84 years: OR 2.60, 95% CI 1.70-3.98, *P*<.001; ≥85 years: OR 3.17, 95% CI 1.95-5.17, *P*<.001) of Internet use for emails/texting exclusively. Compared to non-Hispanic whites, non-Hispanic blacks and other racial/ethnic groups had higher odds (OR 1.60, 95% CI 1.03-2.49, *P*=.04 and OR 2.31, 95% CI 1.33-3.99, *P*=.01, respectively) of using the Internet for emails/texting only. Compared to Internet users with a 4- or 5-year college degree or higher-level education, users with less than a high school education or unknown (don’t know/refused) level of education or a high school education/GED, had higher odds (OR 2.20, 95% CI 1.28-3.79, *P*=.01 and OR 1.61, 95% CI 1.09-2.38, *P*=.02, respectively) of using the Internet for emails/texting only. Those with higher income (in $10,000) had lower odds (OR 0.93, 95% CI 0.89-0.98, *P*=.01) of using the Internet for emails/texting only.

## Discussion

This study examined not only the relationships among Internet use and health needs, psychological capital, and social capital, but also the relationships among different types of Internet use and these variables among a nationally representative sample of community-dwelling older adults aged 65 and older in the United States. Although some previous studies have examined the characteristics of older health information technology users [8,14,16,17,25], this study is one of the first to examine the characteristics of older adults who engage in other types of Internet use activities. The 51% prevalence of Internet use among older adults in 2011 (compared to 58% in the 2012 Pew Internet use survey) lags far behind that of younger adults, including those in the 50 to 64 age group. Nevertheless, older Internet users engaged in diverse types of Internet activities: almost 86% of the users sent emails/text messages, 51% shopped, paid bills, and/or did banking, and 45% conducted health-related tasks on the Internet. Just 9% of the users used the Internet only for sending emails/text messages.

The findings show that demographic and socioeconomic status variables were significant predictors of Internet use versus nonuse. As previous studies demonstrated [14,16-18], black and Hispanic individuals are less likely to use the Internet, and one of the strongest determinants of older adults’ Internet use is their education level. Compared to college graduates, high school graduates were 80% less likely to use the Internet, and those with less than a high school education were 90% less likely to use the Internet. As expected, having a diagnosis of dementia or Alzheimer’s disease significantly lowered the odds of Internet use, but the number of chronic medical conditions (excluding dementia or Alzheimer’s disease) and ADL/IADL impairments were not significant factors. With regard to psychological capital, H1 was supported because both depressive symptoms and anxiety symptoms were associated with a lower likelihood of Internet use, and H2 was not supported because self-efficacy was not related to Internet use. With regard to social capital, the findings support H3 because having both a living child and having a living sibling significantly increased the odds of Internet use. H4 was largely supported because all social integration/tie indicators, except the informal caregiving variable, were significantly associated with Internet use. However, unlike other social capital variables, religious service attendance decreased the odds of Internet use. In general, those who attended religious services were more likely to be women (whose Internet use did not differ from that of men) and non-Hispanic black, Hispanic, and those of other ethnic groups (who are less likely to use the Internet), but attenders also had characteristics associated with higher levels of Internet use, specifically higher levels of education and social capital (eg, more interactions with family and friends, more volunteering, and more participation in clubs, classes, and other organized activities).

The findings also show that different types of Internet users share some similar characteristics, but have different characteristics as well. Having a higher number of medical conditions (implying more health care needs) and engaging in formal volunteering increased the odds of Internet use for health-related tasks, thus supporting H5. Also, H6 and H7 regarding psychological and social capital factors associated with Internet use for various purposes were also partially supported. Anxiety symptoms decreased the odds of Internet use for health-related tasks and increased the odds of email/texting only. Engagement in volunteer work was associated with increased odds of Internet use for health-related tasks, but decreased odds of using the Internet for email/texting only. Religious service attendance was associated with decreased odds of Internet use for shopping/banking tasks. Sociodemographic correlates of using the Internet only for email/texting were the older age groups (80-84 and ≥85 years), a black or other racial/ethnic background, a high school education or less, and lower income. These findings show that older adults who used the Internet for email/texting purposes only were the most socially and economically disadvantaged group of the Internet users.

As discussed, Internet technology can offer multiple benefits and conveniences for older adults dealing with physical and functional decline and social isolation in later life. Owing to advances in Internet and other mobile technology, individuals now can access more information about their health than ever before [19]. The Internet also has the potential to help older adults with disabilities carry out health care-related and other activities with greater ease (eg, without having to rely on others for transportation). This study confirms the findings of previous studies that older adults who are older and socioeconomically disadvantaged are significantly less likely to use the Internet, including accessing health-related information. Given the pervasive Internet technology use among young and middle-aged people, Internet use among future generations of older adults will be common; however, the current generation of older adults who use the Internet, especially those in the older group (≥80 years), learned to use the Internet in late life [6,9]. A previous study found that nonusers were most likely to cite financial reasons for their lack of computer use, specifically the cost of computer equipment and Internet access [19]. The present study suggests that a large proportion of the oldest age group does not use computers/Internet for a variety of reasons, such as lack of financial resources or of social support to do so. Given the decreasing cost of computers/tablets, public or private not-for-profit programs are needed to provide inexpensive devices (eg, netbooks, Chromebooks) and Internet subscriptions for low-income older adults.

Along with sociodemographic variables, this study also found that most social capital variables were significantly correlated with Internet use, as hypothesized. This confirmation of the importance of social integration/ties and social support in facilitating older adults’ learning and adoption of Internet technology also suggests a synergistic relationship: by teaching those with less social capital to use computers and the Internet, their social capital may increase because computer/Internet use can increase their ties to others (relatives, support groups, hobby groups, etc).

The study has a few limitations. First, the NHATS presently offers only a cross-sectional dataset (longitudinal data will be provided in the future); thus, only correlational, not causal, relationships could be deduced. Second, measures of psychological capital used in NHATS—depression, anxiety, and self-efficacy—were abbreviated, not full, scales. The shortened scales may not have adequately captured the complex nature of psychological capital. Moreover, the self-efficacy and anxiety scales were not specific to computer/Internet use. Third, although NHATS provides the most recent data on technology use among a nationally representative sample of US older adults, it did not include a full array of Internet activities in which these older adults may have engaged. Such data would have provided a more valuable description of older adults’ Internet activity. Finally, NHATS did not distinguish email from texting. The distinction may have provided a clearer picture of either activity.

Despite these limitations, this study’s results have significant implications for future research and computer/Internet technology training for older adults. First, research should reexamine the role of psychological capital, especially self-efficacy and anxiety symptoms, using scales that specifically measure psychological capital pertaining to computer/Internet use and using longitudinal data. Second, research should identify characteristics of religious service attenders that may be associated with lower odds of their Internet use, since church settings may provide venues for overcoming barriers to Internet use, teaching computer/Internet skills, and encouraging computer/Internet use. Third, computer/Internet training for older adults needs to consider the significant role other social support networks can play as well. Children, other family members, and friends may rally around older adults who have anxiety about learning Internet technology. A related benefit is that younger people may feel a sense of accomplishment from teaching older adults how to use the Internet or other technology. Intergenerational connections can be established by expanding or developing programs in which high school and college students volunteer at senior programs, assisted living facilities, or in the homes of homebound older adults to teach computer and Internet operational skills, including how to use the Internet for fun and relaxation as well as obtaining health information, making appointments, banking, and other tasks. Fourth, computer/Internet technology training for older adults needs to focus on the older age group of older adults, racial/ethnic minority older adults, older adults with low levels of education and low income, those not married and living alone, and those with low levels of social integration and social support. Older adults with these characteristics can potentially benefit the most from what Internet technology can offer.

Health care sectors are adopting increasing numbers of telehealth and telemental health interventions for older adults [39-41]. With accumulating evidence of their potential to improve access to health and mental health services among geographically and socially isolated older adults and other underserved groups, Internet- and mobile-based health and mental health care service delivery is expected to become a widespread reality in the near future [42,43]. Older adults must be prepared for the changing health care delivery and eHealth services by improving their access to and training for Internet technology. Previous studies have found that older adults with socioeconomic disadvantages were able to learn computer and Internet use to seek health information in collaborative training sessions, and that the participants in training sessions showed a reduction in computer anxiety and increase in computer self-efficacy in retrieving and evaluating online health information [6,44]. Unfortunately, the current study shows that the digital divide is still very real, and that poorly educated, socially isolated, racial/ethnic minority older adults are still not riding the Internet technology wave.

## Abbreviations

ADL: activities of daily living
IADL: instrumental activities of daily living
NHATS: National Aging and Health Trends Study
